# Food-based dietary guidelines for children and adolescents

**DOI:** 10.3389/fpubh.2022.1033580

**Published:** 2022-12-02

**Authors:** Júlia Laura Corrêa Rezende, Maria Carolina de Medeiros Frazão Duarte, Giselle Rhaisa do Amaral e Melo, Luana Caroline dos Santos, Natacha Toral

**Affiliations:** ^1^Nutrition Department, University of Brasília, Brasília, Brazil; ^2^Nutrition Department, Federal University of Minas Gerais, Belo Horizonte, Brazil

**Keywords:** adolescent, child, health promotion, dietary recommendations, scoping review

## Abstract

**Objective:**

This study aimed at reviewing food-based dietary guidelines (FBDGs) with content targeted at children and adolescents to present their main characteristics, thus enabling comparisons among countries.

**Design:**

We conducted a search of the FBDGs available on the Food and Agriculture Organization (FAO) website, followed by a scoping review with a gray literature search to find FBDGs for children or adolescents non-listed on the FAO's website. Data extraction included the year of publication, language, and guidelines for the target group.

**Results:**

From FAO website searches, 109 documents were found, and 17 of them could not be translated. The Scoping review search conducted in 5,190 articles, and none led to new guidelines, nor from the gray literature. Regarding the 92 FBDGs explored, 41 were specific for infants under 24 months old, children, and/or adolescents, and 51 were for the general population with information for the studied group. Twelve percent of the general FBDG and 35% of the specific ones have food icons. All of the guidelines were published after 2001. Latin America and the Caribbean were the regions that presented more specific FBDGs and the majority of countries with guidelines for fruits and vegetables. The information about fat (15 countries) and sugar (26 countries) consumption reduction is frequent. Reduction of sodium intake appears to be in the majority of guidelines after 2015. Food hygiene guidelines are recurrent in Latin American documents. NOVA classification was adopted in five countries and 21 countries approach recommendations for mealtimes. Both exclusive and continued breastfeeding guidance and healthy complementary feeding orientation are present in over 50% of the specific FBDG for infants and children under 24 months old.

**Conclusion:**

Food-based dietary guidelines are diverse due to both the nutritional and political aspects of each region. Latin America stands out for its orientations for the studied group. Further studies should measure the possible impacts and comprehension of FBDGs.

## Introduction

Food-based dietary guidelines (FBDGs) are a practical tool for building a more conscious diet based on healthy and sustainable habits since they provide advice on foods, food groups, and dietary patterns to promote overall health, foster healthy eating habits and lifestyles and prevent chronic diseases (1, 2). They can effectively assist the general population, health professionals, and policymakers in different areas, such as nutrition in public health, agriculture, and nutrition education (2). However, the ways in which these guidelines are presented are diverse, varying between countries and according to the stages of life they are targeted (3, 4).

Specific FBDGs for each stage of life are necessary due to the individualities they present. A healthy eating pattern for each stage of life can demand different habits, which should lead to different dietary guidelines (5). The 1st years of life, especially from 0 to 2 years old, are considered an important window of opportunity as it comprises the formation of eating behavior that will be maintained in adulthood (6). In addition, adolescence has particular characteristics, involving elements of biological growth and major social role transitions, that affect eating patterns in this stage of life (7).

Horta et al. have already conducted a review of FBDGs aimed at children and adolescents (8); however, it is outdated, since it was conducted in 2010. Recently, two systematic reviews compared FBDGs for adults (9, 10), but no other studies focusing on children and adolescents were found.

Thus, this study aimed at reviewing the FBDGs with content targeted at children and adolescents around the world, providing information to policymakers on their main characteristics, in order to enable and assist improvements in the country's tool and comparison among countries.

## Methods

This study was divided into two parts. The first one corresponds to a search conducted on the Food and Agriculture Organization (FAO) website (11): https://www.fao.org/nutrition/education/food-based-dietary-guidelines,—in order to explore the FBDG repository available. This website was launched in 2014 and it has been continually updated. It collates information about FBDG from many countries, including the official name of the national guidelines; publication year; a description of the process and stakeholders involved in its development; the intended audience; a brief description of the food guide; and its key messages. Links to downloadable documents are available too, which were the object of this first part of our study. All FBDGs available were considered, independent of the version, as a complete FBDG, folder, or food guide. “Food guide” and FBDG were considered synonymous. The information was sought not only in materials specifically for children and adolescents but also in those directed to the general population.

Two reviewers (1R and 2R) were responsible for the extraction of FBDG with information for the intended group on FAO's website. In case there was no information for children and adolescents, the material was excluded. For documents whose existence was indicated in FAO's website, but were not available, when there was an indication of the existence of a more recent version of the material in the website, and/or when an earlier FBDG was from previous knowledge of 1R and 2R, a search in official government pages of the respective countries was done. This phase lasted from February 2021 up to March 2021 and it was updated in July 2022 up to August 2022. Guidelines in English, Spanish, Portuguese, French, German, Italian, Chinese, Korean, and Japanese were translated and their information was extracted.

A table used for the extraction of FBDG found on FAO's website was designed based on previous studies (8–10). Information registered of each material found was: whether it was specific or not for children and/or adolescents, country of origin, region (Latin America and the Caribbean, Europe, Asia and the Pacific, North America, Africa, and Near East), language, year of publication, the age group for which the information was intended (infants and children under 24 months old, named group 1; preschoolers and school-age children between 25 months old and 9 years old, named group 2; and adolescents from 10 to 19 years old, named group 3), intended audience (e.g., general population and health professionals), disposition of information in the general FGDBs (e.g., in a specific chapter or annex), and the content directed to the age group. Regarding the content, variables registered were about the presence of a food icon, food groups and/or portion recommendations, recommendations for mealtimes/commensality (e.g., encourages involving the child in preparing meals or eating with the child), and other relevant recommendations, such as fruits and vegetable guidelines, NOVA food classification system, hygiene guidelines, healthy complementary feeding guidelines, recommendation to avoid sugary foods and sweets, fats related information (to limit consumption or about the adequate sources), water ingest, sodium/salt limitation, exclusive breastfeeding, and continued breastfeeding.

NOVA classifies foods into four groups, according to the extent and purpose of the industrial processing they undergo: (1) minimally processed foods and unprocessed food (no addition of salt, sugar, oils or fats, or other food substances to the original food); (2) processed culinary ingredients (like oils and fats, sugar, and salt); (3) processed foods (industrial products made by adding culinary ingredients to the first groups of foods, to increase their durability or enhancing their sensory qualities); and (4) ultra-processed foods (formulations of ingredients, most of exclusive industrial use, which result from a series of industrial processes; products are usually highly profitable, convenient, and hyper-palatable) (12).

The second part of the study corresponds to an additional search in databases following the guidelines of a scoping review in order to check FBDGs for children or adolescents not found on the FAO's website. A scoping review seems to be a more effective way to identify the types of available evidence in a given field (13). This scoping review was written according to the PRISMA-ScR (Preferred Reporting Items for Systematic reviews and Meta-Analyses extension for Scoping Reviews) checklist (14), and the protocol registration is available online under DOI registration, 10.17605/OSF.IO/J5Z6R (15).

A scoping review aiming to identify scientific articles that mentioned FBDGs non-listed in FAO's website for children or adolescents (groups 1, 2, and/or 3) was conducted in March 2021 by two reviewers (1R and 2R). We searched for articles that mentioned guidelines for children and adolescents or the general population with information for children and adolescents, but articles that mentioned FBDGs already identified on FAO's page were not included. Searches were conducted in the following databases: Lilacs, Scielo, PubMed, and Web of Science. This search was complemented by gray literature from Google Scholar (first 100 results). The primary search strategy adopted for the PubMed database (Table 1) was adapted for the other ones. Articles could be in English, Spanish, or Portuguese, without a limit of the year of publication. The risk of bias was not performed since the aim of the study was not to evaluate the included articles. For the entire selection process, the app Rayyan was used (16). If there was a new FGDB identified, the data extraction would be the same as described before.

**Table 1:** Search strategy for PubMed database.

**Database**	**Search strategy**
PubMed	Infant OR Infants OR Baby OR Babies OR “Preschool child” OR “Preschool children” OR Newborn OR Newborns OR “Young child” OR “Young children” OR Child OR Children OR Kid OR Kids OR Toddler OR Toddlers OR “School child” OR “School children” OR Adolescent OR Adolescents OR teenager OR teenagers OR Teen OR Teens OR Adolescence OR Infante OR Infantes OR Niño OR Niños OR Chico OR Chicos OR Bebé OR Escolares OR “Recién nacido” OR “Recién nacidos” OR Adolescente OR Adolescentes OR Criança OR Crianças OR Bebê OR Bebês OR “Pré escolar” OR “Pré escolares” OR “Recém nascido” OR “Recém nascidos” OR Escolar OR Escolares OR Adolescência. AND “Food based dietary guidelines” OR “Food based dietary guideline” OR FBDG OR “Food guide” OR “Food guides” OR “Nutrition Guidelines” OR “Nutrition Guideline” OR “Nutrition policy” OR “Nutrition policies” OR “Nutritional requirement” OR "Nutritional requirements” OR “Nutritional education” OR “Guías alimentarias” OR “Orientación nutricional” OR “Educación nutricional” OR “Necesidades nutricionales” OR “Política nutricional” OR “Necessidades nutricionais” OR “Educação nutricional.”

The materials will be described in a narrative form and with data organized in tables, with no intention of qualifying them.

## Results

**Figure 1:**
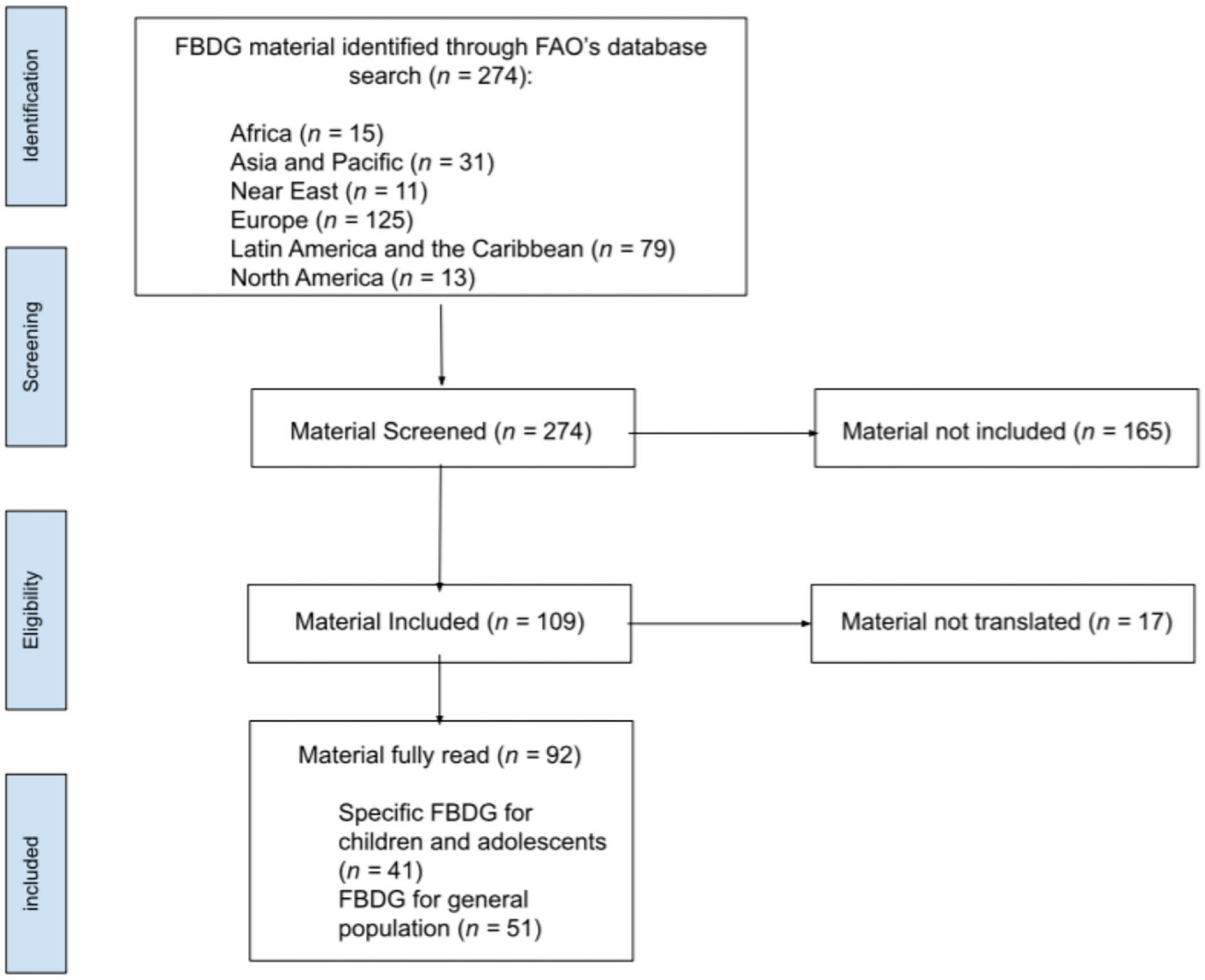
Flow diagram representing the selection process of material from FAO's database.

As presented in Figure 1, from the FAO's page extraction, 274 documents were found, of which 109 had information for children and adolescents. Of them, 17 could not be translated. Regarding the 92 FBDGs explored, 41 (17–57) were specific for children and/or adolescents, and 51 (58–108) were directed to the general population with information targeted at children and/or adolescents.

**Figure 2:**
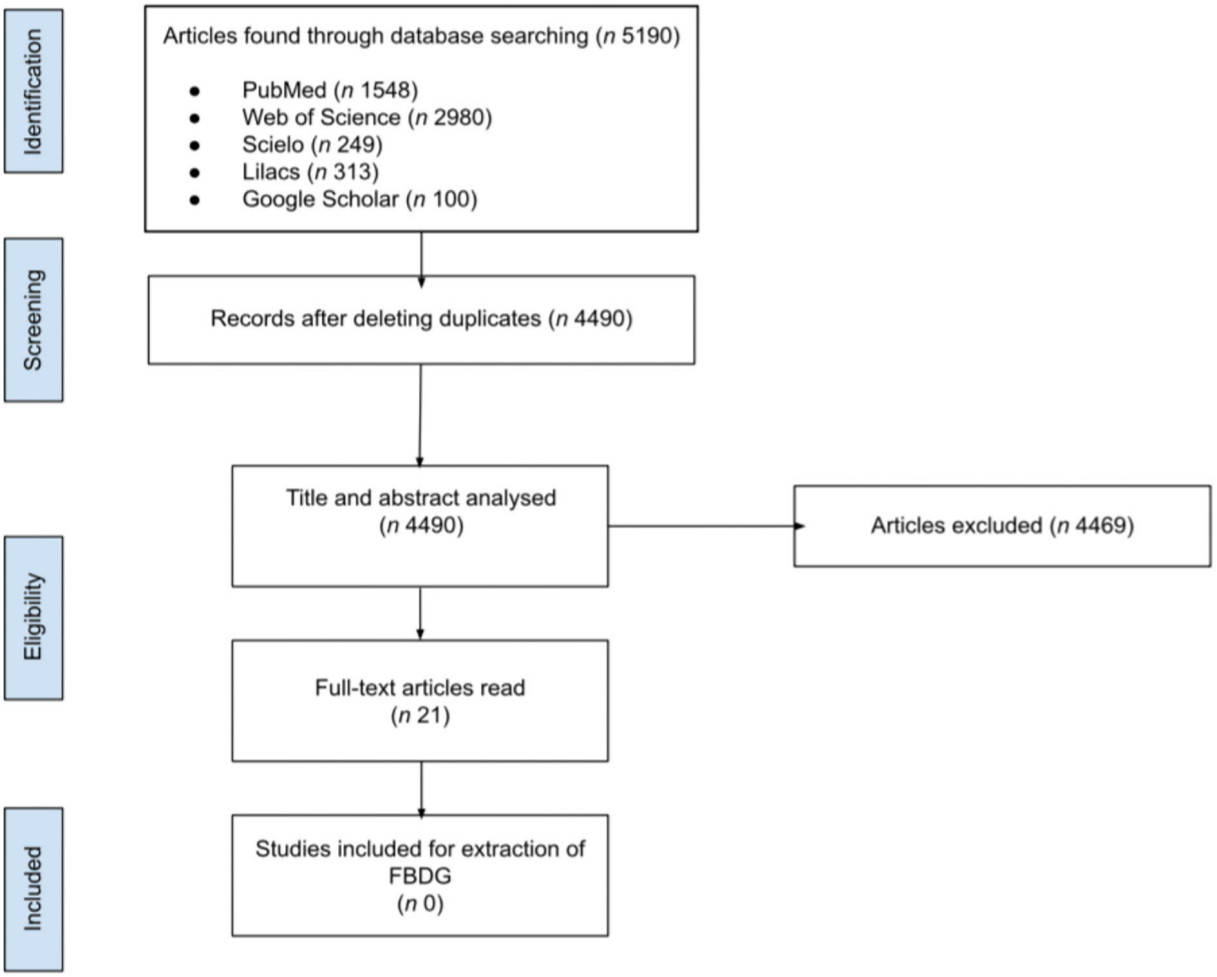
Flow diagram representing the selection process of articles for part 2 (systematic review) of the present study.

As shown in Figure 2, the search for the systematic review resulted in a total of 5,190 articles, of which 700 were duplicates. After the analysis by 1R and 2R, 21 articles were fully read. Neither of the documents were used, nor were the ones found in gray literature, since they mentioned FBDGs for children or adolescents already listed on FAO's website.

### Overview of included material

Ninety-two documents found were from a total of 59 countries, accordingly to the regions listed on FAO's website: 72% of the listed countries in Latin America and the Caribbean (25–44, 81–95), 67% of the five countries in the Near East (76–80), 61% of 18 in Asia and the Pacific (17–24, 66–75), 35% in Europe (45–58, 96–102), all nine countries in Africa (59–65, 105–108), and all two countries in North America (103, 104).

Most of the FBDG documents found were written in English (51%) (17–24, 39, 40, 48, 60–81, 83, 89, 93, 96–98, 100–108), followed by Spanish (31%) (25, 26, 28–38, 41–44, 57, 82, 85–88, 90–92, 94, 95), French (45–47, 49, 50, 59), and German (51–56), representing 7% each, and the remaining documents (4%) were written in Portuguese (27, 84, 99) and Italian (58). Regarding the region of origin, 38% of the documents were from Latin America and the Caribbean (25–44, 81–95), 23% from Europe (45–58, 96–102), 19% from Asia and the Pacific (17–24, 66–75), 12% from Africa (59–65, 105–108), 6% from the Near East (76–80), and 2% from North America (103, 104).

**Figure 3:**
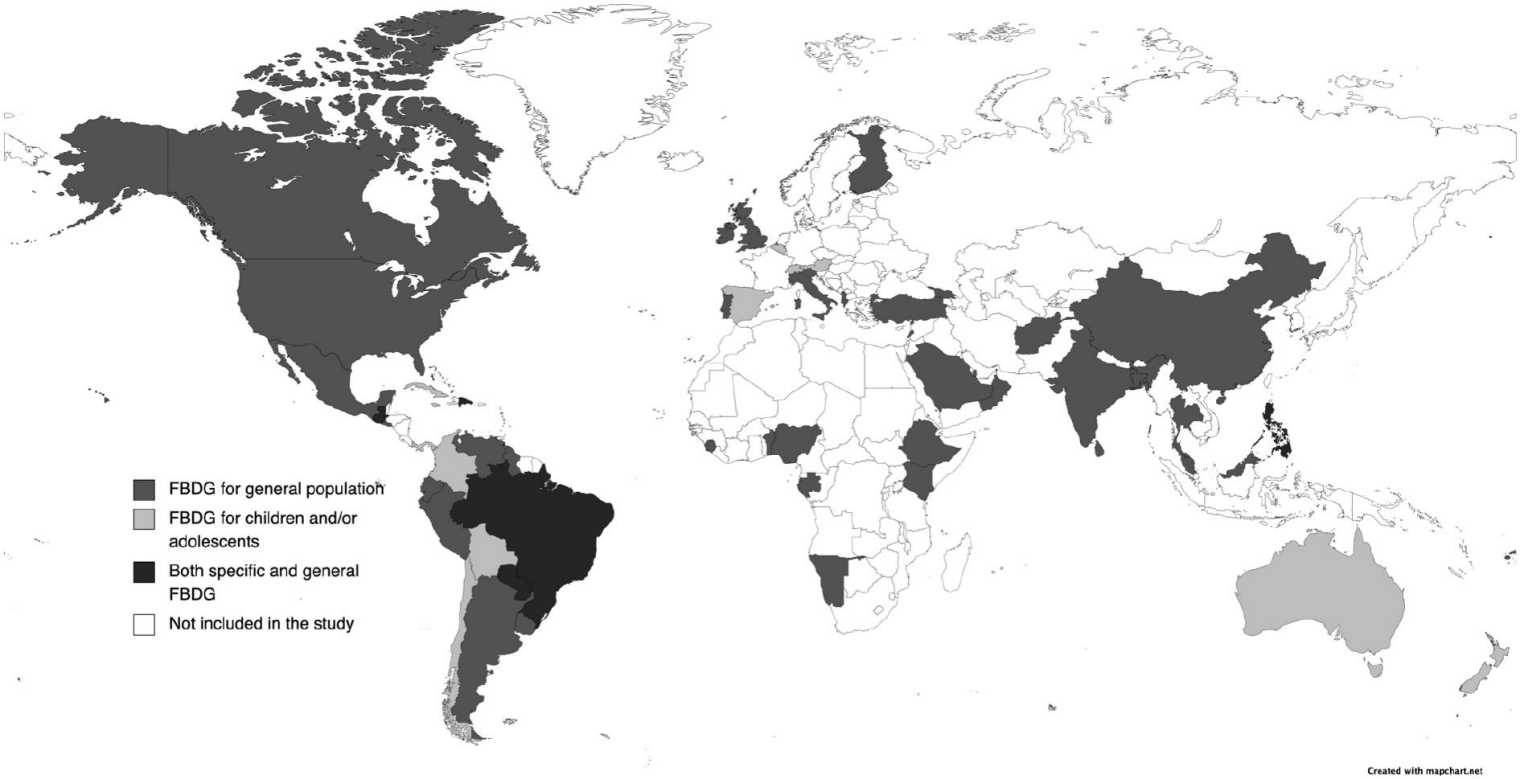
Distribution of FBDG with content targeted at children and adolescents around the world.

About a third (30%) (27, 32, 33, 41, 42, 45–48, 50–56, 58, 82, 86, 89, 92, 97, 103–108) were dated from the past 5 years (2018–2022), 40% (17–26, 28–31, 39, 40, 43, 44, 49, 59, 60, 64–70, 76, 78, 79, 81, 84, 90, 91, 94, 101, 102) were from 2013 to 2017 and 19% (34–37, 71–74, 77, 80, 85, 87, 88, 93, 95, 96) were published between 2008 and 2012. The minority (11%) (38, 57, 61–63, 75, 83, 98–100) are dated before 2008, and none were published before 2000. The distribution of those FBDGs around the world is shown in Figure 3.

### FBDG for the general population with information for children and adolescents

Forty-seven countries with general FBDG with specific information about the studied groups around the world were found: 15 in Latin America and the Caribbean (81–95), two in North America (103, 104), eight in Europe (58, 96–102), nine in Asia and the Pacific (66–75), nine in Africa (59–65, 105–108), and four in the Near East (76–80). Of those, only 12% (58, 69, 70, 85, 97, 102) have food icons directed at children and adolescents. Their main characteristics are summarized in Supplementary Table 1.

The intended age group most frequently addressed in the general FBDG corresponds to group 1 (13%) (63, 72, 74, 81, 83, 89, 108), followed by group 2 (4%) (61, 94). None presented guidelines exclusively for group 3. As for those materials which comprehend more than one group, the majority contains recommendations for groups 1, 2, and 3 (40%) (58, 60, 62, 64, 69, 71, 77–79, 86, 90, 93, 95–101, 104), 33% for groups 2 and 3 (59, 67, 68, 73, 75, 76, 80, 82, 84, 88, 91, 92, 102, 105–107), and 10% for 1 and 2 (65, 66, 70, 85, 87). About the intended audience, most of the FBDG (54%) were targeted at the general population (58, 59, 61–64, 67–71, 73, 75, 77, 80, 81, 83, 87–89, 91, 94, 95, 99, 100, 102, 108), 28% were directed to professionals of a certain field, mainly health (65, 66, 72, 76, 85, 86, 90, 92, 96–98, 101, 103, 104), and 18% were directed for both the general population and professionals (60, 74, 78, 79, 82, 84, 93, 105–107) (Supplementary Table 1).

Most of the documents (57%) (58–62, 64–66, 71, 73–75, 77, 80, 87, 88, 90, 91, 95–98, 100–102, 104–107) presented food groups directed for the target population of this study. Nineteen percent (60–62, 65, 69, 90, 95–98) have recommendations for mealtimes, directed at children and adolescents (Supplementary Table 1).

### Specific FBDG for children and adolescents

Seventeen countries with specific FBDG for the studied group were found: 59% in Latin America and the Caribbean (25–44), 23% in Europe (45–57), and 18% in Asia and the Pacific (17–24). None was found in North America, Africa, and the Near East. From those 17 countries, 35% (23–26, 32, 33, 45–56) have specific icons for children and adolescents. The characteristics of those FBDGs for children and adolescents are summarized in Supplementary Table 2.

About half of the specific FBDG were aimed at group 1 (24%) (27, 34–38, 41–44), followed by group 2 (10%) (28–31) and group 3 (5%) (39, 40), and documents intended for more than one age group: 39% (17–22, 45–47, 51–57) corresponds to groups 1, 2, and 3, 12% (32, 33, 48–50) to groups 1 and 2, and 10% (23–26) to groups 2 and 3. Sixty-four percent (17–22, 25, 26, 28–31, 34–37, 41–50) were organized in guidelines and 32% (23–26, 28–31, 38–42) did not present recommendations for mealtimes. From the studied material that presents food groups, 85% (17–22, 27–38, 41–57) present at least four food groups: cereals or similar, animal protein sources, milk and dairy, and fruits and vegetables (Supplementary Table 2).

**Figure 4:**
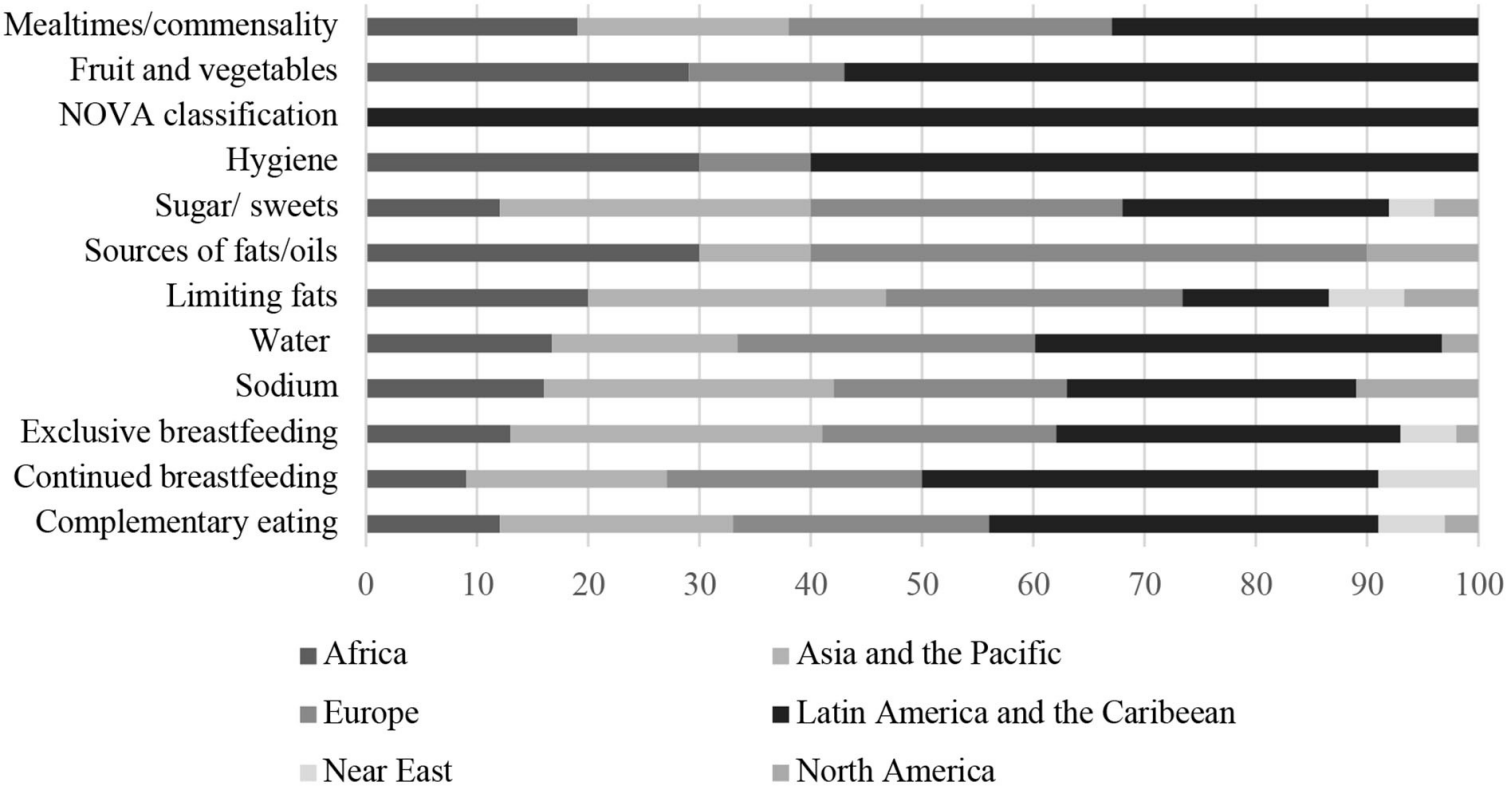
Percentage distribution of FGDBs with specific nutritional recommendations according to world regions.

**Figure 5:**
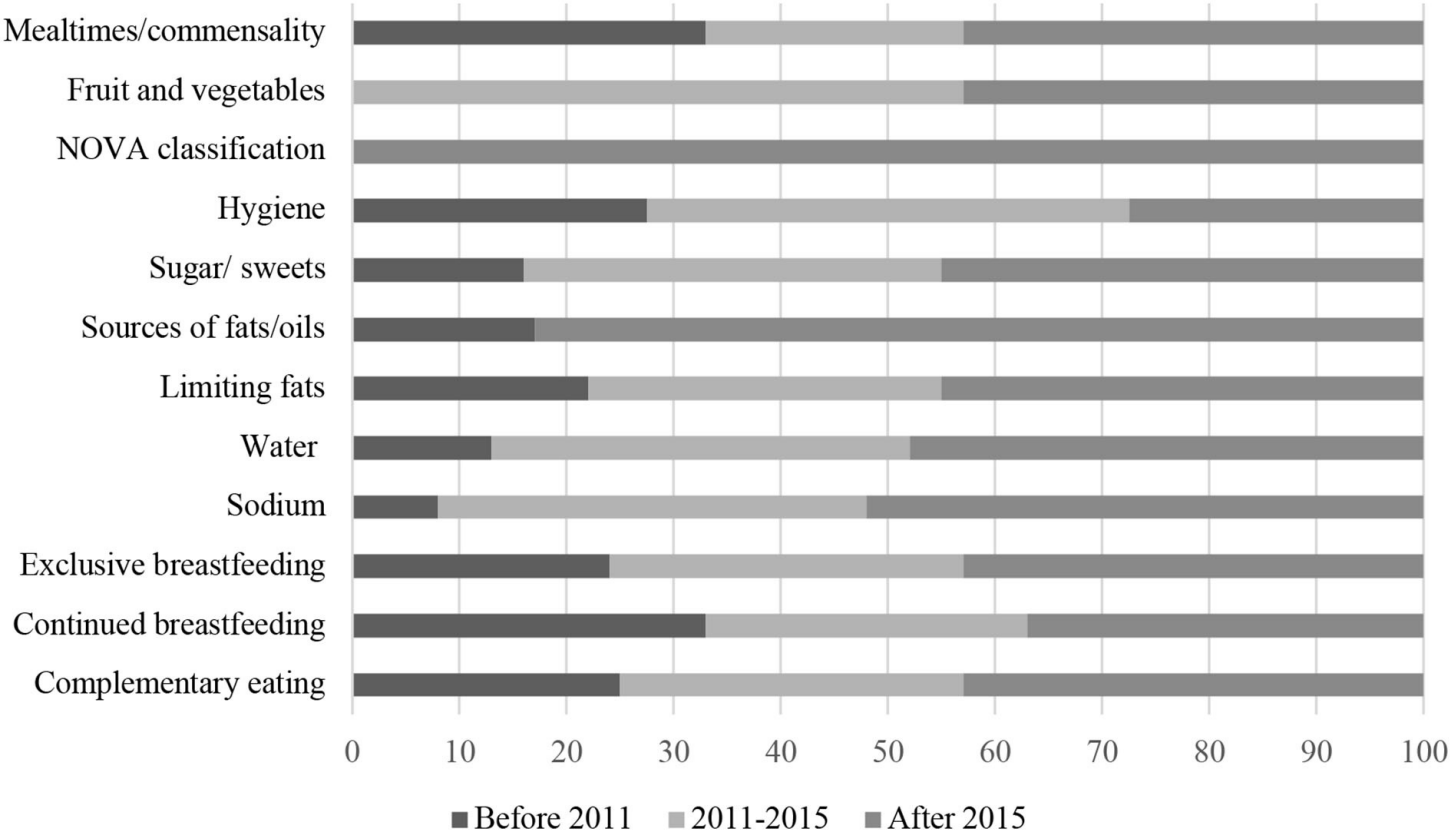
Percentage distribution of FGDBs with specific nutritional recommendations according to the year of publication.

Figures 4, 5 show the distribution of relevant specific recommendations found in the FBDGs, according to world regions and year of publication, respectively.

**Figure 6:**
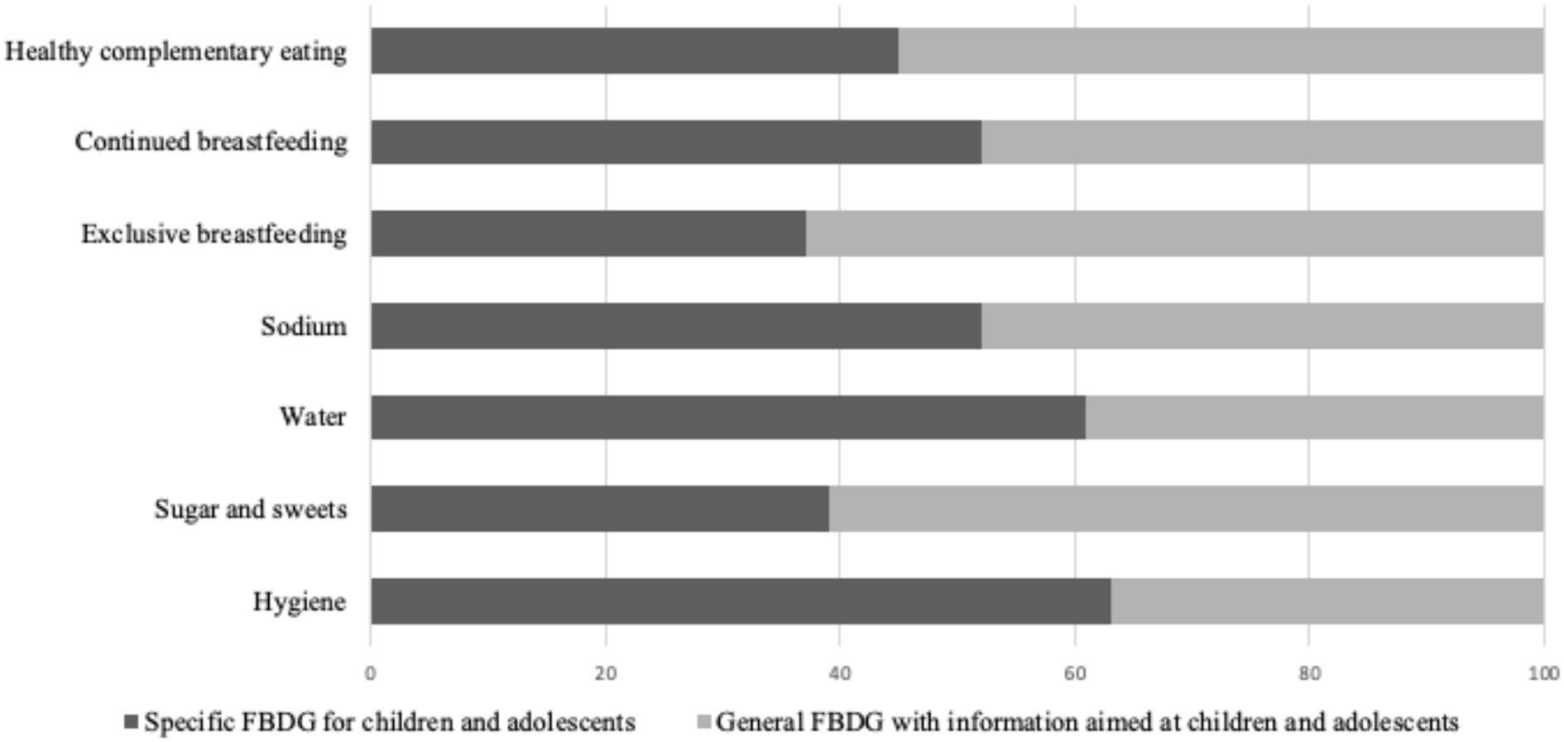
Percentage distribution of FBDGs with specific nutritional recommendations according to the type of document.

**Figure 7:**
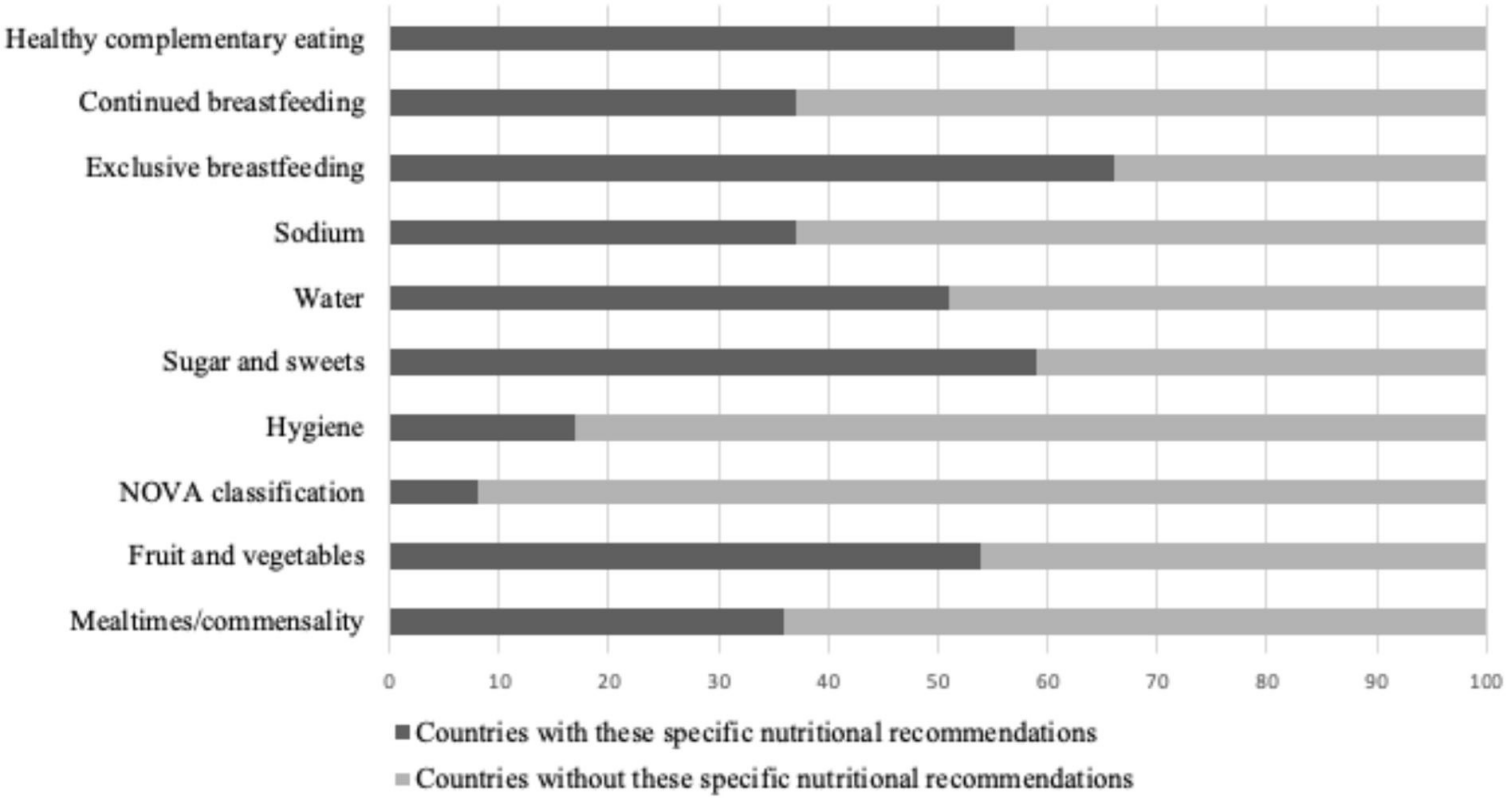
Percentage distribution of countries with specific nutritional recommendations in their FBDGs.

Figure 6 shows the distribution of important specific recommendations found in the FBDGs according to the type of document (general or specific for the studied age groups) and Figure 7 presents, in percentage, the number of countries with these specific recommendations in their FBDGs.

#### Recommendations for mealtimes and commensality

It was found 21 out of the 59 countries included guidance for eating habits in mealtimes, such as eating with the family, without using cellphones or television, in a calm environment (17, 21, 27, 32, 34, 37, 43, 45, 50, 53, 60–62, 65, 69, 90, 96–98). From these 21, 19 FBDG included guidance for eating habits in mealtimes for children and/or adolescents. There was no significant variation between specific and general documents on this topic (Supplementary Table 2).

#### Fruit and vegetables

Of the 27 countries, whose national FBDG had dietary guidelines aimed at children and adolescents, seven have an exclusive guideline for fruit and/or vegetable consumption (25, 26, 28–30, 49, 60, 65, 90), 67% of those documents were specific FBDG (25, 26, 28–30, 49). Also, 32 countries out of the 59 analyzed in this study bring some guidance about fruit consumption on the FBDG (17, 19–22, 25, 26, 28–31, 35, 48, 49, 57–60, 62, 64–67, 69, 71, 74, 75, 77, 80, 87, 90, 91, 96, 104–107), 43% were specific documents for children and adolescents (17, 19–22, 25, 26, 28–31, 35, 48, 49, 56, 57), and 26 encourage or guide vegetable consumption for the studied group (17, 20, 22–31, 35, 48, 57–60, 62, 64–66, 69, 71, 74, 75, 77, 90, 91, 96, 105–107), 45% were in specific FBDG for the target population of this study (17, 20, 22–31, 35, 48, 57).

#### NOVA classification

Five (8%) out of the 59 countries showed the NOVA food classification system to gather the foods in groups in the FBDG, according to the processing level (27, 84, 86, 87, 92, 94). These countries are all from Latin America: Brazil, Ecuador, El Salvador, Peru, and Uruguay. Of the six FBDGs, which included the NOVA classification, most of them (83%) were general FBDGs (83, 85, 86, 91, 93).

#### Sugar and sweets

Among the 35 countries in which FBDG includes sugar and sweets guidance for children and adolescents, 26 (74%) showed guidance to reduce or avoid its consumption (17–22, 27, 29, 38–41, 49, 52, 58, 60, 64, 65, 67, 70, 71, 74, 75, 80, 85, 96–98, 101, 104–107). Of the 33 FBDG that provided this information, about a third (39%) were specific documents for the studied group (17–22, 27, 29, 38–41, 49, 52).

Besides the recommendations for reducing or avoiding sugar consumption, 11 countries (32%) (25, 26, 32–35, 41–44, 58, 69, 71, 90, 91, 100, 101) brought guidance on the number of portions or grams of sugar per day, accordingly to age. As for the minimum age to be introduced to sweets, 20% (20–22, 41, 42, 48, 49, 58, 74, 97, 100) recommended 12 months old, 17% (17–19, 27, 60, 96, 101, 104), 24 months old, and 1 (65) country advised to not offer sugar to children under 3 years old. Some of the materials also correlated high sugar consumption with chronic diseases, such as diabetes, corresponding to 6% (86, 105–107) of the 35 countries. One country (45, 49) presented the recommendation to introduce sugar for 8 months old children.

Approximately, 15% (64, 67, 97, 104–107) of the countries recommended sugar intake below 10% of total daily calories, of those two (64, 105–107) countries limit sugar intake to 5% of daily.

More than half (65%) (17, 25, 28, 29, 31, 34, 35, 41, 42, 48, 49, 57, 60, 64, 65, 69, 71, 74, 75, 90, 91, 96, 97, 100, 101, 104–107) of the countries presented some information about sugar and sweets intake addressed for age group 2 (preschool-age and school-age children), 53% (18–20, 27, 41–45, 48, 49, 60, 64, 65, 71, 74, 96, 97, 100, 101, 104–107) for age group 1 (infants and children under 2 years old), and 44% (17, 26, 30, 31, 34, 35, 39, 40, 60, 75, 90, 91, 96–98, 100, 101, 104) for age group 3 (Adolescents).

#### Fats

From the 33 countries whose FBDG included fat and oils consumption guidance (17–22, 25–32, 34, 35, 37, 38, 43, 45, 49, 56–58, 60, 62, 64–66, 71, 74, 75, 78, 79, 88, 90, 91, 95–98, 100, 101, 104), 10 (30%) provided guidance on the best sources (22, 27, 45, 49, 60, 62, 64, 96, 97, 104), 15 (45.5%) highlighted the limit of fat consumption (17, 19, 21, 22, 49, 56, 60, 64, 65, 69, 75, 80, 85, 88, 98, 101, 104), and 6 (18.2%) guided good sources and limit the consumption of oils and fats (22, 49, 56, 60, 64, 104). Among the 44 FBDGs that provided fat and oils consumption guidance, 55% were specific documents for the studied group (17–22, 25–32, 34, 35, 37, 38, 43, 45, 49, 56, 57, 101).

#### Sodium

Among the 22 countries that the FBDG provides guidance on sodium consumption for the age group studied, 19 focus on limiting its consumption (17–22, 25–27, 33, 41, 45, 56, 58, 60, 64, 65, 71, 74, 75, 84, 95, 97, 103, 104). There were 25 FBDGs that included guidance for sodium consumption for children and/or adolescents, 52% of them were specific FBDGs for the studied group (17–22, 25–27, 33, 41, 45, 56).

About a third (36%) (58, 60, 64, 96, 97, 101, 103, 104) of these countries brought recommendations per age, limiting sodium intake and 23% (20–22, 32, 33, 41, 42, 58, 60) discourages its intake for children under 12 months old. Five countries (17, 60, 65, 74, 104) provide orientation in their FBDG about avoiding salty snacks and foods rich in sodium and one country (94) recommended avoiding having salt on the table at mealtimes.

As for the guidance, about half (45%) (26, 58, 60, 64, 74, 75, 96, 97, 101, 104) of the countries presented information addressed for age group 2 (preschool-age and school-age children), 45% (26, 58, 60, 64, 74, 75, 96, 97, 101, 103, 104) for age group 3 (Adolescents) and 36% (20–22, 27, 32, 33, 58, 60, 97, 101, 104) for age group 1 (infants and children under 2 years old).

#### Exclusive breastfeeding

It was found that 39 countries had exclusive breastfeeding until 6 months old guidance in their FBDGs (18–20, 27, 31, 33, 34, 36, 38, 41–43, 45, 49, 51, 52, 58, 60, 62–74, 78, 80, 81, 89, 90, 95–98, 100, 104, 108). Among the 43 FBDG that provided this information, 37% were specific documents for the studied group (18–20, 27, 31, 33, 34, 36, 38, 41–43, 45, 49, 51, 52). The majority (70%) (27, 34, 36, 38, 41–43) of specific FBDG aimed at group 1 presented guidance about exclusive breastfeeding.

#### Continued breastfeeding

Twenty-two countries have also continued breastfeeding orientation in their FBDG (18, 19, 27, 33, 34, 36–38, 41–43, 51–53, 58, 62, 64, 68, 72, 74, 78, 80, 90, 95–99). Of the 27 FBDGs that provided this information, 52% were specific documents for the studied group (18, 19, 27, 33, 34, 36–38, 41–43, 51–53). About 80% (27, 34, 36–38, 41–43) of specific FBDG directed to group 1 have this recommendation.

#### Healthy complementary eating

Thirty-four countries had FBDG that addressed guidance on healthy complementary eating (18–20, 27, 31, 33, 34, 37, 38, 41, 43, 45, 48, 51–53, 57, 58, 60, 62, 64–67, 69, 71, 72, 74, 78, 80, 88–90, 95–98, 100, 105). There were 38 documents that included guidance for healthy complementary eating, 45% of them were specific FBDG for the studied group (18–20, 27, 31, 33–35, 38, 41, 43, 45, 48, 51–53, 57). More than half (60%) (27, 34, 37, 38, 41, 43) of the materials specific to group 1 presented guidance on this subject.

## Discussion

This article intends to fill a gap about the main characteristics of the current general and specific FBDGs around the world that bring guidance aimed at children and adolescents. A previous study (8) identified 17 specific FBDGs for children and adolescents around the world, so the growing elaboration of this type of material can be seen, given that the present study identified 41 FBDGs aimed specifically at this age group. This can be explained by the relevance that the food habits in childhood and adolescence have in dietary patterns through adulthood and in the child's development (6, 109, 110).

It can also be noticed that the regions that had the highest growth of countries with specific FBDGs since 2011 (8) were Latin America and the Caribbean, with 10 new specific FBDGs since 2011 (25–44). That growth is in line with the context of the double burden of malnutrition that Latin America and the Caribbean countries are experiencing. In this way, the focus of nutrition policies shifts from an undernourished population to a developing country with overweight and obese population (111, 112).

Even though there was an increase in the number of specific FBDGs aimed at children and adolescents around the world, it is not yet widespread in many countries, which is explicit in this study as most of the documents found were aimed at the general population. This shows a limitation to adapt the contents of the guidelines in order to make them understandable and motivational to the target population (113). Also, specific documents are important as each life stage has its singularities, such as nutritional needs and interests (114).

Age groups most cited in general FBDGs are schoolers and preschoolers. This is justified as most countries that have specific documents have those types of guides only aimed at children under 2 years old (20, 27, 32, 34–38, 41–45, 48, 51–53). Thus, it is clear that groups 2 and 3 are neglected, as they also need specific nutritional recommendations and the number of specific materials for each of them is fewer (18, 19, 21–26, 28–31, 33, 39, 40, 46, 47, 54–56).

When analyzing the results obtained by the study, it is possible to notice the great differences in the way of presenting nutritional guidance for the studied group, both in specific and general FBDGs. This information can be exposed in the documents through nutritional guidelines, icons, didactic design, food groups, and others. Those differences can be explained by the singularities of each region, the prevalence of nutritional inadequacies, and food culture, e.g., China's food icon being an abacus (69, 115).

Food icons or graphic representations are a tool for nutritional education, as they can represent quantities and even the frequency of consumption recommended for each food group in a succinct way (115). There are some differences between the shapes of food icons found. There is often a relation between the countries' cultures and the shapes chosen, as it seems to evoke the cultural food choices and some cultural food elements suggest cooking, besides the proportion between food groups (116). It is important to remember the relevance of culture in food choices since food practices such as cooking and having meals can be part of identity (117).

However, literature has suggested limitations when it comes to FBDGs' graphic representations, such as Food Pyramids and Plates, due to the attempt to summarize all choices consumers need to make in order to maintain healthy eating habits (118). Another important aspect is that the interpretation of such elements can demand subjective comprehension and the possible struggle of the population on comprehending abstract concepts (119).

About food groups, most of the specific FBDG presented a meat/animal source protein. When it comes to recommendations, developing countries with high undernutrition prevalence recommend red meat consumption in order to prevent anemia, especially among younger groups (9), such as Guatemala's FBDG (38). Others recommend altering between protein sources, which is an important recommendation and seems to be related to environmental sustainability concerns, such as Panama's document (41), Brazil's (27), China's (69), Belgium's (46, 47), and Australia's materials (17, 19), in which the recommendations were to consume other protein sources, such as lean meats and poultry, besides vegetable protein sources as legumes and nuts.

Another feature explored related to diet quality is commensality. Eating in the company of the family has proved to have a protective effect against obesity in children and adolescents, besides supporting healthy eating habits. This practice elevates the consumption of fruits and vegetables and several micronutrient intakes. It also decreases the risk of being overweight and obese in adolescents (120). Therefore, it is valuable that the FBDG present recommendations to endorse family meals as recommendations for mealtimes, both general and specific FBDG (17–22, 27, 36, 37, 45–56, 60, 65, 90, 95, 96).

Asia and the Pacific are the regions with most countries with FBDG that brought guidance on fruit (29%) (17, 19–22, 66, 67, 71, 74, 75) and vegetable (32%) (17, 20, 22–24, 66, 69, 71, 74, 75) consumption, which might demonstrate concern with the population's eating habits such as the growth rates of ultra-processed food consumption (121).

It is observed that Latin America is in a food transition, characterized by lower consumption of fruits and vegetables and with a high or growing participation of ultra-processed products in diet (108). This can justify why most countries with specific guidelines for fruit and/or vegetable consumption were in Latin America and the Caribbean (25, 26, 28–30, 90). It is also related to the nutritional transition presented in these countries, in which there are growing rates of overweight, obesity, and chronic diseases associated with weight gain (111). All of these FBDGs have been published after 2011, 43% after 2015, which is related to the fact that both food and nutritional transitions in Latin America are still considered recent processes.

All FBDG which use the NOVA food classification system were from Latin American countries (39, 40, 53, 54, 68, 70), which can be explained by the development of this tool being made in Brazil, by researchers of the Center for Epidemiological Research in Nutrition and Health of the University of São Paulo (12). Two recent systematic reviews (122, 123) have highlighted an association between high ultra-processed food intake and a variety of adverse health outcomes for adults, such as overweight, obesity, and different non-communicable chronic diseases, including cancer, hypertension, diabetes, and dyslipidemia. Among children and adolescents (123), the outcomes of high ultra-processed food consumption include cardio-metabolic risks and asthma; thus, there is already a body of evidence supporting the incorporation of the NOVA classification in dietary guidelines as a scientific concept to evaluate the “healthiness” of foods, including those directed to children and adolescents. Knowledge about the processing level of food is needed in order to design effective nutritional guidance to prevent chronic diseases and to promote adequate food production and distribution systems (111).

In developing countries, it can be noticed that food-related illness such as diarrhea has an expressive role in children's mortality rates (124). Also, a study showed that there is a substantial correlation between the Human Development Index (HDI) of a country and diarrhea-associated deaths among children (125). Thus, developing countries with lower HDI, such as those in Latin America (25, 26, 34, 37, 41, 43, 90) and Africa (61, 64, 65), represent almost all the countries with hygiene guidelines in the FBDG, to guide the population and prevent those diseases. Also, this explains the lack of a hygiene guideline in FBDGs in regions with developed countries with higher HDI, such as those from North America and Europe.

As most of the materials analyzed that brought orientations on topics such as hygiene and water consumption were specific FBDGs (17–31, 34, 38–41, 43, 45, 47, 49, 51, 52, 56, 57), it is also evident that specific documents can include more about topics indirectly related to food consumption. It is possible because there is space to embrace those topics in specific documents as it provides guidelines for a narrower target audience than general FBDGs.

By 1999, the United States Department of Agriculture (USDA) determined a limitation in the consumption of fats for children, which might explain no FBDG with such guidance before 2001. Also, there is an evident limitation of the FBDGs in relation to the consumption guidelines for the groups of fats and sugars, as this often occurs through recommendations of “moderate use” or “minimum quantity,” which can lead to different interpretations of the amount that should or can be consumed (113).

The World Health Organization (WHO) recommendations for sugar intake are up to 5% of daily calorie intake (126), which was present in 6% (64, 105–107) of the countries that advised about sugar and sweets consumption for children. Dietary patterns rich in sugar can lead to oral caries, diabetes, and other non-communicable diseases (NCDs) (126); therefore, guidance on high-sugar foods and beverages should be present in FBDG (127).

Furthermore, the rising prevalence of obesity and non-communicable diseases in childhood and adolescence is concerning. According to WHO (127), some strategies to prevent obesity are related to limiting the consumption of foods and beverages high in fat, sugar, and salt by infants and young children. Guidance to avoid those foods and regulations on the marketing and sale of beverages and snacks of that category are measures that can be adopted by the government to prevent excess weight gain among children.

Guidance on sodium consumption is recent, which can be noticed as most of the FBDG analyzed with this orientation have been published after 2015 (21, 22, 27, 33, 41, 45, 56, 58, 60, 64, 97, 103, 104). This characteristic can be associated with the greater accumulation of scientific evidence about the harmful effects of excessive sodium consumption and the increasing salt or sodium consumption data among the studied age group (128). Guidance on sodium consumption for children and adolescents is necessary because of the growing pace of pathogenic processes of chronic diseases in these stages of life (129). Research demonstrates that 80% of Brazilian adolescents consume above the upper level of sodium and almost 10% of them have hypertension (128). Sodium consumption has an impact not only in the economic sphere, in relation to diseases associated with excessive consumption, but it is also related to premature death (130).

Yet, reducing salt intake is related to increasing population health, by preventing outcomes such as cardiovascular diseases, besides being a low-cost measure. On this path, it is essential to maintain the population's awareness of the necessity to reduce salt consumption, such as by not having salt shakers on the table at mealtimes, and avoiding high in sodium snacks and foods (131).

Among the 39 countries that presented exclusive breastfeeding guidance, 17 did not mention continued breastfeeding orientation in their FBDGs (20, 31, 45, 49, 61, 64, 66–68, 70–72, 74, 82, 90, 100, 104, 108), recommended by the WHO for the practice to be continued for up to 2 years old or longer (132). As known in the literature, breast milk contains all nutrients to promote the healthy growth and development of infants (133). Besides, a study has shown a negative correlation between breast milk intake with the consumption of ultra-processed food and sweetened beverages, being evident in this impact on childhood obesity and NCDs (134). It seems this type of orientation could reinforce the practice among breast feeders and health professionals; it is, therefore, suggested that updates in the FBDG take this into account.

The study has some limitations regarding the translation of some documents, which were left out such as those in Russian, Khemer, and Hebrew. Also, there might be FBDG not mentioned in the FAO; however, a systematic review was carried out to maximize the possibility of identifying them, but no additional material was found.

## Conclusion

The present study summarized different countries' official recommendations for children and adolescents in order to compare and acknowledge the available content in this field. It was possible to notice the materials' diversity, due to both the nutritional and political aspects of each region. In this context, Latin America stands out for its orientations for the studied group. The relevance of understanding the tendencies around the world is to be aware of possible gaps, without putting aside the specificities of each population. This review did not aim to measure the possible impacts and comprehension of FBDGs, nor other subjective evaluations of the materials, which can be explored by further studies.

## Author contributions

JC and MM: parts 1 and 2 of the study, writing, data extraction, and analysis. GM: data extraction, writing review, and advising/orientation. LS: writing review. NT: design of the study, writing review, and advising/orientation. All authors contributed to the article and approved the submitted version.

## Conflict of interest

The authors declare that the research was conducted in the absence of any commercial or financial relationships that could be construed as a potential conflict of interest.

## Publisher's note

All claims expressed in this article are solely those of the authors and do not necessarily represent those of their affiliated organizations, or those of the publisher, the editors and the reviewers. Any product that may be evaluated in this article, or claim that may be made by its manufacturer, is not guaranteed or endorsed by the publisher.
