# Supplementary material for: Food-based dietary guidelines for children and adolescents
Source: Front Public Health. 2022 Dec 2;10:1033580. doi: 10.3389/fpubh.2022.1033580 (PMC9755327; doi:10.3389/fpubh.2022.1033580)
Supplement: Supplementary file 1 [file Data_Sheet_1.docx]

Supplementary Table 1. Main characteristics of general FBDGs analyzed.

| **Country**  **(Language and year)** | **Age group (yo/mo*)/ Group**** | **Intended audience** | **Disposition of information** | **Food groups and portion recommendations** | **Food icon** | **Recommendations for mealtimes** |
| --- | --- | --- | --- | --- | --- | --- |
| Itália  (Italian, 2018)^(57)^ | 0-3 yo; 1-2 yo; 2-3 yo; 4-6 yo; 7-10 yo; 11-14 yo; 15-17 yo.  Group: 1, 2 and 3 | General population | Along with information for other age groups, a specific table for portions directed for children and adolescents. | 18 food groups based on nutrients with portion recommendations for each age. | Pie chart | Not presented. |
| Benin  (French, 2015)^(58)^ | 2-3 yo; 4-8 yo; 9-13 yo; 14-18 yo.  Group: 2 and 3 | General population | Along with information for other age groups in tables. | 5 food groups based on nutrients with portion recommendations for each age. | None for this age group. | Not presented. |
| Kenya  (English, 2017)^(59)^ | 0-6 mo; 6-23 mo; 24-59 mo; 5-9 yo; 10-19 yo.  Group: 1, 2 and 3 | General population, policy makers, program designers and implementers of healthy diets and physical activity programs, nutritionists, other health practitioners, community educators, agriculture extension workers and teachers. | Chapter for each age-group, from birth to elderly. | 16 food groups based on nutrients with no portion recommendation. | None for this age group. | Children: no pressure for eating and in a calm environment.  Adolescents: eat with the company of the family, avoid screens while eating. |
| Namibia  (English, 2000)^(60)^ | >2 yo.  Group: 2 | General population | Mixed with information for other age groups, in a descriptive way. | 4 food groups based on nutrients with no portion recommendation. | None for this age group. | 3 big meals a day, with healthy snacks between. |
| Nigeria  (English, 2006)^(61)^ | 0-6 mo; 6-12 mo; 12-24 mo; 25-60 mo (2-5 yo); 6-11 yo; 12-18 yo.  Group: 1, 2 and 3 | General population | Each chapter separated in age-groups from birth to elderly. | 5 food groups based on nutrients with no portion recommendation. | None for this age group. | Calm environment, no pressure for eating and the caregiver must be patient. |
| Seychelles (English, 2006)^(62)^ | 0-6 mo.  Group: 1 | General population | Organized in guidelines with no distinction of age-group. One guideline directed for infants. | No division in food groups presented. | None for this age group. | Not presented. |
| Sierra Leone (English, 2016)^(63)^ | Babies and small children, children 2-5 yo; school-age children (6-12 yo); teenagers (12-18 yo).  Group: 1, 2 and 3 | General population | Separate annex for each age-group. | 6 food groups based on nutrients with no portion recommendation. | None for this age group. | Not presented. |
| South Africa (English, 2013)^(64)^ | 0-6 mo; 6-12 mo; 12-36 mo; 3-5 yo.  Group: 1 and 2 | Health professionals and policy makers | Specific pediatric chapter. | 9 food groups based on nutrients with no portion recommendation. | None for this age group. | Correlates parental styles and feeding styles. Encourages a responsive feeding, a calm environment, no pressure for eating, eating with the child. |
| Afghanistan (English, 2016)^(65)^ | 0-6 mo; 6 mo (complementary feeding); 7-8 mo; 9-11 mo; 12-24 mo.  Group: 1 and 2 | Health professionals, teachers, agriculture agents | A couple of the guidelines are directed for children. | 7 food groups based on nutrients with portion recommendations for each age. | None for this age group. | Not presented. |
| Bangladesh (English, 2013)^(66, 67)^ | Children and adolescents; no age specification.  Group: 2 and 3 | General population. | Information for the aimed group among other information. | No division in food groups presented. | None for this age group. | Not presented. |
| China (English, 2016)^(68)^ | 7-24 mo; 2-5 yo; 6-17 yo.  Group: 1, 2 and 3 | General population. | Specific chapters for age groups. | 6 food groups based on nutrients with portion recommendations for each age. | Abacus | Set a schedule for meals, the children must feed themselves and be involved in preparing the meals, 3 meals a day. |
| Fiji (English, 2013)^(69)^ | Group: 1 and 2 | General population. | Guidelines directed for the studied group. | No division in food groups presented. | Pineapple | Not presented. |
| India (English, 2011)^(70)^ | 6-12 mo; 1-3 yo; 4-6 yo; 7-9 yo; 10-12 yo; 13-15 yo; 16-18 yo.  Group: 1, 2 and 3 | General population. | Specific annex for infants, children and adolescents. | 9 food groups based on nutrients with portion recommendations for each age. | None for this age group. | Not presented. |
| Malaysia (English, 2010)^(71)^ | 6 mo-2 yo.  Group: 1 | Nutrition educators, health providers and policy makers | Guidelines divided in chapters. 1 guideline directed for the aimed group. | No division in food groups presented. | None for this age group. | Not presented. |
| Philippine (English, 2012)^(72)^ | 3-5 yo; 6-9 yo; 10-12 yo; 13-18 yo.  Group: 2 and 3 | General population. | Organized in guidelines, 1 of them is for the studied group. | 3 food groups (energy giving; body building; body regulating) based on effect in the body with portion recommendations for each age. | None for this age group. | Not presented. |
| Sri Lanka (English, 2011)^(73)^ | 6-12 mo.  Group: 1 | Health workers and general population | Organized in guidelines, 2 of them are directed for children and adolescents. Each guideline is described in a chapter. | 5 food groups based on nutrients with portion recommendations for each age. | None for this age group. | Not presented. |
| Thailand (English, 2001)^(74)^ | 6-13 yo; 14-25 yo.  Group: 2 and 3 | General population | Recommendations for caloric ranges, classified per age. The ranges are not separated in different chapters. | 6 food groups based on nutrients with portion recommendations for each age. | None for this age group. | Not presented. |
| Lebanon (English, 2013)^(75)^ | Children and adolescents; no age specification.  Group: 2 and 3 | Policy makers, health care providers, dietitians, nutritionists | Information for the studied group spread in the document. | No division in food groups presented. | None for this age group. | Not presented. |
| Oman (English, 2009)^(76)^ | 1-5 yo; 6-14 yo; 14-18 yo.  Group: 1, 2 and 3 | General population. | Portions size and recommendation in tables, separated for age groups. | 6 food groups based on nutrients with portion recommendations for each age. | None for this age group. | Not presented. |
| Qatar  (English, 2015)^(77, 78)^ | Children and adolescents; no age specification.  Group: 1, 2 and 3 | General population, health professionals and policy makers | Organized in guidelines which are divided in chapters. One guideline directed for the aimed group. | No division in food groups presented. | None for this age group. | Not presented. |
| Saudi Arabia (English, 2012)^(79)^ | 2-3 yo; 4-8 yo; 9-13 yo; 14-18 yo.  Group: 2 and 3 | General population | Information for the studied group spread in the document. | 5 food groups based on nutrients with portion recommendations for each age. | None for this age group. | Not presented. |
| Antigua and Barbuda  (English, 2013)^(80)^ | 0-6 mo.  Group: 1 | General population | Organized in guidelines. One guideline directed for the studied group. | No division in food groups presented. | None for this age group. | Not presented. |
| Argentina (Spanish, 2020)^(81)^ | Children and adolescents; no age specification.  Group: 2 and 3 | General population over 2 years-old and any food-based education agents. | Information for the studied group spread in the document. | No division in food groups presented. | None for this age group. | Not presented. |
| Bahamas (English, 2002)^(82)^ | No age specification.  Group: 1 | General population. | Organized in guidelines. One guideline directed for the studied group. | No division in food groups presented. | None for this age group. | Not presented. |
| Brazil (Portuguese, 2014)^(83)^ | Children and adolescents; no age specification.  Group: 2 and 3 | General population and health professionals. | Information for the studied group spread in the document. Influence of the media directed for children and adolescents. | No division in food groups presented for this age group. | None for this age group. | Not presented. |
| Dominican Republic (Spanish, 2009)^(84)^ | Children and infants (0-6 mo and <6 mo).  Group: 1 and 2 | Health Professionals. | Information for the studied group spread in the document. Information about complementary feeding, consumption of fruits and vegetables and breastfeeding. | No division in food groups presented for this age group. | Pestle of nutrition. | Not presented. |
| Ecuador (Spanish 2020)^(85)^ | Children and adolescents; no age specification.  Group: 1, 2 and 3 | Health Professionals | Information for the studied group spread in the document. | No division in food groups presented. | None for this age group. | Not presented. |
| El Salvador (Spanish, 2012)^(86)^ | Children; no age specification.  Group: 1 and 2 | General population | Information for the studied group spread in the document. | 5 food groups based on nutrients with no portion recommendation. | None for this age group. | Not presented. |
| Guatemala (Spanish, 2012)^(87)^ | No age specification. Children and adolescents.  Group: 2 and 3 | General population. | Information for the studied group spread in the document. | 3 food groups for children and adolescents based on nutrients with intake frequency recommendation. | None for this age group. | Not presented. |
| Guyana (English, 2018)^(88)^ | < 6 mo  Group: 1 | General population | Organized in guidelines. One guideline directed for the studied group. | No division in food groups presented. | None for this age group. | Not presented. |
| Mexico (Spanish, 2015)^(89)^ | 0-6 mo; 6-24 mo; 2-12 yo; 13-18 yo  Group: 1, 2 and 3 | Health Professionals | Specific chapters with information directed for the studied group. | 5 food groups based on nutrients with portion recommendations for each age. | None for this age group. | Eat in company, in a calm environment and with no pressure for eating. |
| Paraguay  (Spanish, 2015)^(90)^ | Children and adolescents, no age specification.  Group: 2 and 3 | General population | Information for the studied group spread in the document. 2 guidelines mention children and adolescents. | 1 food group with intake recommendation for the age group. | None for this age group. | Not presented. |
| Peru (Spanish, 2019)^(91)^ | Children and adolescents; no age specification.  Group: 2 and 3 | Health professionals and policy makers | Information for the studied group spread in the document. | No division in food groups presented. | None for this age group. | Not presented. |
| Saint Kitts and Nevis (English, 2010)^(92)^ | Children and adolescents; no age specification.  Group: 1, 2 and 3 | Health professionals, policy makers, community leaders, educators and the general population | Information for the studied group spread in the document. | No division in food groups presented. | None for this age group. | Not presented. |
| Uruguay (Spanish, 2016)^(93)^ | Children; no age specification.  Group: 2 | General population | Information for the studied group spread in the document. | No division in food groups presented. | None for this age group. | Not presented. |
| Venezuela (Spanish, 2010)^(94)^ | Children and adolescents; no age specification.  Group: 1, 2 and 3 | General population | Information for the studied group spread in the document. | 3 food groups based on nutrients with no portion recommendation. | None for this age group. | Eat in family |
| Albania (English, 2008)^(95)^ | Children and adolescents; no age specification.  Group: 1, 2 and 3 | Health professionals | Specific chapters with information directed for the studied group. | 10 food groups based on nutrients with portion recommendations for each age range. | None for this age group. | Eat with company, switch off TV while eating |
| Finland (English, 2019)^(96)^ | Children and adolescents; no age specification.  Group: 1, 2 and 3 | Health Professionals | Information for the studied group spread in the document. | 7 food groups based on nutrients with portion recommendations for each age range. | Plate | Eat in company and health habits. |
| Georgia (English, 2005)^(97)^ | 0-1 yo; 1-3 yo; school-age children; adolescents (girls: 11-15; boys: 13-16)  Group: 1, 2 and 3 | Health Professionals | Specific chapters with information directed for the studied group. | 8 food groups based on nutrients with no portion recommendation. | None for this age group. | Children must sit calmly while eating. |
| Portugal (Portuguese, 2002)^(98)^ | Children and adolescents; no age specification.  Group: 1, 2 and 3 | General population | Information for the studied group spread in the document. | No division in food groups presented. | None for this age group. | Not presented. |
| Turkey (English, 2006)^(99)^ | 0-1 yo; 1-3 yo; 4-6 yo; 7-9 yo; 10-18 yo.  Group: 1, 2 and 3 | General population | Information for the studied group spread in the document. | 4 food groups based on nutrients with portion recommendations for each age range. | None for this age group. | Not presented. |
| UK (English, 2016)^(100)^ | 1-18 yo  Group: 1, 2 and 3 | Health Professionals | Specific tables for each age group. | Carbohydrates, Protein and Fat. Portion recommendations for each age range. | None for this age group. | Not presented. |
| Ireland (English, 2016)^(101)^ | 5-12 yo; 13-18 yo  Group: 2 and 3 | General population | Specific recommendations per age. | 6 food groups based on nutrients with portion recommendations for each age range. | Food Pyramid | Not presented. |
| Canada (English, 2019)^(102)^ | Children and adolescents; no age specification.  Group: 2 and 3 | Health Professionals and policy makers | Information for the studied group spread in the document. | No division in food groups presented. | None for this age group. | Not presented. |
| USA  (English, 2020)^(103)^ | 0-6 mo; 6-12 mo; 1-2 yo; 2-18 yo.  Group: 1, 2 and 3 | Health Professionals | Specific chapters with information directed for the studied group. | 6 food groups based on nutrients with portion recommendations for each age range. | None for this age group. | Not presented. |
| Ethiopia  (English, 2022)^(104, 105,106)^ | Children >2yo and adolescents.  Group: 2 and 3. | General population, health and agriculture professionals, school teachers and interested in promoting healthy diets | Specific tables for each age group. | 6 food groups based on nutrients with portion recommendations for each age range. | None for this age group. | Not presented. |
| Gabon  (English, 2021)^(107)^ | Infants (< 6mo).  Group: 1. | General population. | One key-message/  guideline. | No division in food groups was presented. | None for this age group. | Not presented. |

*years old/months old

**group 1: infants and children until 24 months-old ; group 2: preschoolers and school-age children (25 months-old up to 9 years-old); group 3: adolescents (10 to 19 years-old).

Supplementary Table 2. Main characteristics of specific FBDG analyzed.

| **Country**  **(language and year)** | **Age group (yo/mo*)/**  **Phase **** | **Intended audience** | **Food groups and portion recommendations** | **Food icon** | **Recommendations for mealtimes** | **Organized in Guidelines** |
| --- | --- | --- | --- | --- | --- | --- |
| Australia  (English, 2013)^(16, 17, 18)^ | 2-3 yo; 4-8 yo; 9-11 yo; 12-13 yo; 14-18 yo  Group: 1, 2 and 3 | General population and health professionals | Vegetables and legumes/beans; fruits; grain (cereal) foods; lean meats and poultry, fish, eggs, tofu, nuts and seeds, and legumes/beans; milk and dairy products.  Portion recommendations of food groups for each age groups. | None for this age group. | Turn off the TV while eating and have meals with the family. | Only for phase 2 and 3:  1- To achieve and maintain a healthy weight, be physically active and choose amounts of nutritious food and drinks to meet your energy needs.  2- Enjoy a wide variety of nutritious foods from these five food groups every day:  3- Limit intake of foods containing saturated fats, added salt, added sugars and alcohol.  4- Encourage, support and promote breastfeeding.  5- Care for your food; prepare and store it safely. |
| New Zealand  (English, 2013 - phase 1; 2017 - phase 2; 2019 - phase 3)^(19, 20, 21)^ | 0-2 yo; 2-12 yo; 13-18 yo  Group: 1, 2 and 3 | General population | Phase 1: breads, fruits and vegetables; salad vegetables; milk and dairy products; meats/egg; cereals, nuts and seeds.  Phase 2: breads and cereals; fruits and vegetables; lean meats, chicken, seafood, eggs, legumes, nuts and seeds; milk and dairy products.  Portion recommendations of food groups for each age groups. | None for this age group. | Have meals in family, with TV off. No pressure on the child to eat. | Phase 1:  1- Breast milk is best.  2- If you are not breastfeeding, use an infant formula until the baby is 12 months old.  3- Give babies and toddlers plenty to drink.  4- Start solid food around 6 months old.  5- Change the variety, texture and quantity of food as your baby grows.  6- Healthy eating habits start early.  7- Once your baby starts solids try to have some meals together as a family.  Phase 2:  1- Make mealtimes fun.  2- Have meals together as a family and turn off the TV and cell phones.  3- Have meals at times that suit children.  4- Provide three healthy meals every day, including breakfast.  5- Provide a wide variety of healthy foods for children to choose from.  6- Encourage your child to choose healthy foods, such as fruit and vegetables.  7- Encourage children to try new foods.  8- Involve children from an early age to prepare foods and let them do more as they get older.  9- Make children’s serving sizes smaller than an adult’s.  10- Encourage family members to stop eating when they feel full.  11- Offer healthy snacks between meals.  12- Keep takeaways for occasional meals only.  13- Limit fruit juice and dried fruit.  14- Don’t use rewards or force or push a child to eat.  15- Don’t encourage continuous eating or grazing.  Phase 3:  1- to grow and be healthy, you need to be active, eat the right foods and get enough sleep.  2- Make healthy food choices; eat many different foods;  3- Always take time to eat a healthy breakfast; 4- Keep some fruit and  a bottle of tap water  in your bag in case you get hungry or thirsty,  5- Have plenty to drink,  6- Sit less, Move more, Sleep well. |
| Philippines (English, 2016)^(22, 23)^ | 3-12 yo; 13-18 yo  Group: 2 and 3 | General population | Rice and alternatives, fish and alternatives, vegetables, fruits. Portion recommendations of food groups for each age group. | Plate | Not presented. | Not organized in guidelines |
| Bolívia  (Spanish, 2013)^(24, 25)^ | 6-7 yo; 8-10 yo;  11-18 yo  Group: 2 and 3 | Health Professionals | School-age children: cereals, tuber and legumes; vegetables; fruits; milk and dairy products; fats; sugar and sweetened products.  Adolescents: milk and dairy products; cereals; legumes; tuber and roots; vegetables; fruits; oils and fats; sugar. Portion recommendations of food groups for each age groups. | “Food Arch” | Not presented. | 1- Varied diet with all food groups, increasing intake of fruits and vegetables.  2- Increase milk and dairy products intake.  3- Consume sources of Iron (meats) at least 3 times a week.  4- Choose vegetal oils.  5- Use iodized salt but with moderation  6- Ingest 6-8 glasses of water a day.  7- Avoid exaggerated consumption of sugar, sweets and alcohol.  8- Reduce coffee and tea intake and consume milk and fruit juice.  9- 30 minutes of physical activities a day.  10- Wash your hands before mealtime. |
| Brazil  (Portuguese, 2019)^(26)^ | 6-12 mo; 12-24 mo.  Group: 1 | Health Professionals | Cereals; roots and tubers; vegetables; fruits; nuts and seeds; milk and cheese; meats and egg; water  +  Nova classification: In Natura; Processed food; Ultraprocessed food.  No portion recommendations of food groups. | None for this age group. | 1- Eat with company  2- Turn off the TV at mealtimes  3- Offer a calm environment for children to eat  3- No pressure on child for eating  4- No offering treats associated to meals | Not organized in guidelines. |
| Chile  (Spanish, 2018)^(27, 28, 29, 30)^ | 2-5 yo; 6-10 yo.  Group: 2 | Health Professionals | Milk and dairy; vegetables; fruits; chicken and meat; legumes; eggs; cereals, pasta or potatoes; breads; oils and fats. Portion recommendations of food groups for each age group. | None for this age group. | Not presented. | Phase 2: 1- Make eating fruits and vegetables a habit for your child;  2- Pay attention to label and nutritional facts of the foods and choose the ones that contain less fats, sugar and salt;  3- Encourage your child being active;  4- Reduce screen time to less than 2 hours a day;  5- Make sure the children get 8 hours of sleep. |
| Colombia  (Spanish, 2018 - phase 1; 2019 - phase 2)^(31, 32)^ | 0-2 yo; 2-5 yo.  Group: 1 and 2 | Teachers, first-level health personnel, agricultural extension workers, officials of government entities, NGOs, journalists. | Cereals, tubers, roots; fruits and vegetables; milk and dairy products; meat, eggs, legumes and dried fruits; fats; sugar. No portion recommendations of food groups. | Plate | No screen use during mealtimes. | Not organized in guidelines. |
| Cuba  (Spanish, 2009)^(33, 34)^ | 0-2 yo  Group: 1 | Health Professionals | Cereals; vegetables; fruits; meats, chicken, fish, eggs and beans; milk, yogurt and cheese; fats; sugar. No portion recommendations of food groups. | None for this age group. | “Don’t force your child to eat, be patient and offer a calm and no distraction environment.” | 1- Exclusive breastfeeding until the baby is 6 months-old.  2- Breastfeeding is an act of love.  3- At 6 months-old, introduce complementary feeding.  4- Offer natural foods, without added sugar or salt  5- After 6 months-old, offer meat and chicken to children frequently. After 8 months-old, offer fish and seafood.  6- After 1 year-old, babies can eat the family food.  7- Be careful with preparing foods, be hygienic.  8- Don’t force your child to eat, be patient and offer a calm and no distraction environment. |
| Dominican Republic (Spanish, 2009)^(35, 36)^ | 0-6 mo; 6-23 mo.  Group: 1 | Health Professionals | Cereals; legumes; fruits; vegetables; food from animal sources; fats; sugar; salt. No portion recommendations of food groups. | None for this age group. | 1- Eat with company  2- Turn off the TV at mealtimes  3- Offer a calm environment for children to eat | Phase 1:  1- Keep breastfeeding up to 2 years or more;  2- Encourage children to complementary feeding;  3- Be hygienic when manipulating or preparing food;  4- Increase the offer of food as the child grows but keep breastfeeding;  5- Evolve the amount and consistency of foods as the child grows;  6- Increase frequency of meals as the child grows;  7-Natural foods are healthier;  8- Offer natural foods without adding salt or sugar;  9- Use natural spices  10- Combine legumes with cereals, cook them until the grains are soft and the broth is dense;  11- Teach your children to brush their teeth after mealtime. |
| Guatemala (Spanish, 2003)^(37)^ | 0-1 yo.  Group: 1 | Health Professionals | No food groups division and no portion recommendations. | None for this age group. | Not presented. | Not organized in guidelines. |
| Jamaica (English, 2015)^(38, 39)^ | Adolescents, no age specification  Group: 3 | Adolescents | Fruits; fats and oils; food from animals; legumes and nuts; staples; vegetables. No portion recommendations of food groups. | None for this age group. | Not presented. | Not organized in guidelines. |
| Panama (Spanish, 2018)^(40, 41)^ | No age specification  Group: 1 | Parents and Health professionals | No food groups division and no portion recommendations. | None for this age group. | Not presented. | 1- Exclusive breastfeeding in the first 6 months of life.  2- Offer a variety of foods from 6 months-old.  3- Prepare foods without salt or sugar.  4- 1 year-old must eat all healthy foods along with their family.  5- Offer the child meat, chicken, eggs, fish and seafood.  6- Feed your child frequently, encourage him to eat without pressure.  7- Washing hands and manipulating foods hygienically can keep your child healthy.  8- Play with your child every day. |
| Paraguay (Spanish, 2015)^(42, 43)^ | 0-6 mo; 6-24 mo  Group: 1 | Health professionals and policy makers. | Fruits; milk and dairy products; sugar and honey; oils and fats; meats; legumes; eggs; vegetables; cereals and tubers. Portion recommendations of food groups for each age group. | None for this age group. | 1- Turn off the TV at mealtimes  2- Offer a calm and clean environment for children to eat | 1- Exclusive breastfeeding up to 6 months-old.  2- Initiate complementary feeding at 6 months-old, with smashed foods.  3- At 1 year-old, the child must eat family food.  4- Encourage children to eat fruits and vegetables.  5- Feed your child in a clean and calm environment, be patient and caring.  6- Consuming soda, sweetened beverages and sweets can lead to tooth decay and obesity.  7- Food and hand hygiene helps your child to keep healthy.  8- In case of diarrhea, keep feeding your child to prevent weight loss. |
| Belgium (French, 2020)^(44, 45, 46)^ | 0-3 yo  6-12 mo  12-18 yo  Group: 1, 2 and 3 | Pregnant Women and parents of children 0-3 yo.  Teenagers. | 7 food groups based on nutrients, with portion recommendations for 0-3 yo. | Food Pyramid | Family meals. | Infants:  1- Orientation for children without appetite;  2- Family meals.  3- Offer 4 meals: breakfast, lunch, snack and dinner. For breakfast, offer bread, fruit, milk or dairy and a beverage (juice without added sugar, milk or water), and for a snack, a dairy product, a beverage of choice, a cereal and a fruit.  Phase 1:  1- Breastfeeding and child formula orientation.  Phase 3:  1- Consume tubers and cereals for energy;  2- Tips for healthy eating at school, restaurants and at home;  3- Increase water intake;  4- Girls must pay attention to Iron intake and both boys and girls must consume calcium, vitamin D and protein;  5- Alcohol in adolescence is not recommended;  6- Eating healthy leads to a healthier skin. |
| Switzerland (French, 2017/2018)^(47, 48, 49)^ | 0-3 yo  Group: 1 and 2 | Parents of children 0-3 yo. | Beverage; fruits and vegetables; cereals, tubers and legumes; milk and dairy products; meat, fish, eggs and tofu; fat and nuts; snacks; sweets. No portion recommendations of food groups. | Pie chart | 1- Eat with company  2- No pressure on child for eating  3- No offering treats  associated to meals  4- Mindful eating, no screen at mealtimes. | 1- Breastfeeding and child formula orientation, complementary feeding as well. 2- Avoid beverages with caffeine.  3- Excess of sugar can lead to tooth decay.  4- Information on supplements and allergies.  5- Orientation for children without appetite.  6- Pay attention to nutritional facts and labels of the products offered to children 2-3 years-old.  7- Add vegetables in meals, such as meats, rice to increase nutrients. |
| Áustria (German, 2020)^(50, 51, 52, 53, 54, 55)^ | 0-1 yo; 1-3 yo; 4-10 yo.  Group: 1, 2 and 3 | Parents. | No food groups division and no portion recommendations. | Food Pyramid | 1- Eat with company  2- Turn off the TV at mealtimes  3- Offer a calm environment for children to eat  4- No pressure on child for eating  5- No offering treats  associated to meals | Not organized in guidelines. |
| Spain (Spanish, 2005)^(56)^ | Children and adolescents; no age specification.  Group: 1, 2 and 3 | Health Professionals and general population | 13 food groups based on nutrients with portion recommendations for each age range. | None for this age group. | Not presented. | Not organized in guidelines. |

*years old/months old

** group 1: infants and children until 24 months-old; group 2: preschoolers and school-age children (25 months-old up to 9 years-old); group 3: adolescents (10 to 19 years-old).
